# Toll-Like Receptor and Cytokine Responses to Infection with Endogenous and Exogenous Koala Retrovirus, and Vaccination as a Control Strategy

**DOI:** 10.3390/cimb43010005

**Published:** 2021-04-30

**Authors:** Mohammad Enamul Hoque Kayesh, Md Abul Hashem, Kyoko Tsukiyama-Kohara

**Affiliations:** 1 Transboundary Animal Diseases Centre, Joint Faculty of Veterinary Medicine, Kagoshima University, Kagoshima 890-0065, Japan; mehkayesh@pstu.ac.bd (M.E.H.K.); mdhashem29@yahoo.com (M.A.H.); 2 Department of Microbiology and Public Health, Faculty of Animal Science and Veterinary Medicine, Patuakhali Science and Technology University, Barishal 8210, Bangladesh; 3 Department of Health, Chattogram City Corporation, Chattogram 4000, Bangladesh; 4 Laboratory of Animal Hygiene, Joint Faculty of Veterinary Medicine, Kagoshima University, Kagoshima 890-0065, Japan

**Keywords:** koala, koala retrovirus, toll-like receptors, cytokines, immune response, vaccines

## Abstract

Koala populations are currently declining and under threat from koala retrovirus (KoRV) infection both in the wild and in captivity. KoRV is assumed to cause immunosuppression and neoplastic diseases, favoring chlamydiosis in koalas. Currently, 10 KoRV subtypes have been identified, including an endogenous subtype (KoRV-A) and nine exogenous subtypes (KoRV-B to KoRV-J). The host’s immune response acts as a safeguard against pathogens. Therefore, a proper understanding of the immune response mechanisms against infection is of great importance for the host’s survival, as well as for the development of therapeutic and prophylactic interventions. A vaccine is an important protective as well as being a therapeutic tool against infectious disease, and several studies have shown promise for the development of an effective vaccine against KoRV. Moreover, CRISPR/Cas9-based genome editing has opened a new window for gene therapy, and it appears to be a potential therapeutic tool in many viral infections, which could also be investigated for the treatment of KoRV infection. Here, we discuss the recent advances made in the understanding of the immune response in KoRV infection, as well as the progress towards vaccine development against KoRV infection in koalas.

## 1. Introduction

The koala (*Phascolarctos cinereus*), an iconic marsupial of Australia, is facing severe population decline due to man-made hazards and natural infections [1,2]. Koala retrovirus (KoRV) is considered to be one of the major infectious agents in koala populations impacting koala health both in the wild and in captivity [3,4,5,6,7,8,9,10,11]. The replication-competent full genome sequence of KoRV was reported in 2000 [12]. KoRV, a single-stranded positive-sense RNA virus, belongs to the Retroviridae family and the *Gammaretrovirus* genus, with a genome of 8.4 kb containing gag, pol, and env genes, and an integrated KoRV provirus additionally contains long terminal repeats at both ends [4,12,13]. KoRV has the typical morphology of a gammaretrovirus, which consists of spherically shaped virions ranging between 80 and 100 nm in diameter [14]. Recently, KoRV gained the interest of many virologists due to its unique feature of existing in both exogenous and endogenous forms, providing a unique opportunity to gain insights into how retroviruses infect their new host and evolve together in the early stages of genome invasion [15].

Although endogenous retroviruses (ERVs) are ubiquitous features of mammalian genomes, in contrast to other mammalian species the endogenization process of KoRV is a relatively recent event, having started approximately 22,000 to 49,000 years ago or more recently, and is assumed to be still ongoing [13,16]. Several studies have indicated the existence of ongoing retroviral invasion of the koala germline [15,16,17]. Evolutionarily, KoRV and the gibbon ape leukemia virus are very closely related [18], and other close relatives of KoRV include feline leukemia virus (FeLV) [14], porcine endogenous retrovirus (PERV) [14], and melomys burtoni retrovirus (MbRV) [19]. In terms of transmission, KoRV shows some resemblance to other retroviruses. KoRV infection is a recent trans-species transmission event in koalas [20]; however, from which species the virus jumped into koalas is yet unknown. A native Australian rodent, the grassland melomys (*Melomys burtoni*) has been considered as one of the candidate species [18,19], and recently bats were also suggested [21]. Until now, 10 different KoRV subtypes (KoRV-A to KoRV-J) have been reported, where KoRV-A is assumed to exist as both endogenous and exogenous forms, and the other subtypes (KoRV-B to KoRV-J) are considered to be exogenous [10]. KoRV-A and KoRV-B have been characterized most extensively; however, the other subtypes largely remain to be characterized [2,22]. The entry receptors utilized by KoRV-A and KoRV-B are different, where KoRV-A uses a phosphate transporter (PiT1) [23], and KoRV-B uses thiamine transporter 1 (ThTR1) [5,24]; however, the entry receptors for the other subtypes (KoRV-C to KoRV-I) have not been identified. As reported previously, KoRV prevalence varies based on the geographical location of a particular population [25]. As observed, KoRV-A has 100% prevalence in northern Australian koala populations, indicating that KoRV-A has been fully endogenized in northern koalas [22]. However, there is little or no evidence for endogenization in southern koala populations, but the prevalence of KoRV is on the increase in southern koalas [22,26,27]. In a recent study, Hobbs et al. reported defective KoRV-D and KoRV-E subtypes in koalas with significant deletions in the gag and pol genes [15]. ERV and exogenous retrovirus (XRV) interactions by recombination may generate ERV-XRV chimeras [28]. Recently, many recombinant KoRVs (recKoRV) were reported in koalas [15,29,30]. In addition, Löber et al. showed that recombination with an ancient koala retroelement disables KoRV, which frequently occurs at an early point in the invasion process [29].

Different evolutionary, epidemiological, immunological, and clinical aspects of KoRV infection in koala populations have been reviewed in several previous studies [2,14,20,22,31,32,33,34,35,36]. The understanding of KoRV is rapidly growing, and therefore in this review we aimed to focus on the recent advances of host–virus interactions, vaccine development, and the investigation of alternative strategies for protecting koala health and conservation.

## 2. Health Effects of KoRV on Koalas

Although the direct evidence for KoRV regarding the disease’s occurrence in koalas is yet to be established because of the complex nature of the virus that exists in both endogenous and exogenous forms [37], it is widely assumed that the integration of KoRV into the koala genome has effects on koalas’ immune responsiveness and disease susceptibility [14]. Moreover, neoplasia and immunosuppressive disorders are known to be caused by retrovirus infections [38,39]. An increasing number of in vitro and in vivo studies suggest KoRVs as potent immunomodulators that modulate the transcription of immune-related genes [40,41,42,43].

It has been reported that lymphoid neoplasms are linked to high KoRV viral loads, and they are observed at a higher rate in northern koala populations [33,44,45]. Lymphoma was reported for the first time in a South Australian KoRV-A-positive koala in 2017 [7], and recently [44] with increased KoRV proviral and viral loads. Among the exogenous KoRV subtypes, KoRV-B has been reported as more pathogenic, and has been associated with leukemia in koalas [5]. Waugh et al. reported that KoRV-B infection is associated with chlamydial disease in wild koala populations [6]. Butcher et al. reported that southern koalas were more likely to present with periodontitis, which might be due to the exogenous koala retrovirus infection, which might facilitate the development of periodontitis by the modulation of the immune response to concurrent oral bacterial infections [46].

A recent study reported an upregulation of oncogenes in a human cell line (HEK293T) infected with KoRV [47]. In another study, McEwen et al. showed an association of KoRV in cancer development in koala by characterizing KoRV integration sites (IS) in healthy and tumor tissues, and found the dysregulation of genes containing IS and a highly-expressed transduced oncogene [48]. Accordingly, they suggest that the germline invasion of KoRV may confer a tremendous mutational load to the host and may contribute towards cancer development [48]. A thorough transcriptome analysis in koala infected with endogenous and exogenous KoRV subtypes is of great importance to gain more insights into how KoRV is affecting koala health, and the ways to prevent or control it.

## 3. Innate Immune Response to KoRV Infection in Koalas

The innate immune response is a key component of host defense which shapes adaptive immunity. The innate immune response is critical for the hosts, and acts as the first line of immune defense in many viral infections [49]. Viral nucleic acids and proteins can be recognized by different pattern recognition receptors (PRRs) such as Toll-like receptors (TLRs), RIG-I-like receptors, and NOD-like receptors [50]. Unfortunately, the innate immune response characterization in koala is just beginning to scrape the surface, which might be linked to the unavailability or limited availability of koala-specific reagents and characterization tools (antibodies, PCR, etc.). However, the recent availability of genetic resources for koala [30,51] might enhance the development of characterization tools that should greatly facilitate immune response characterization in this species.

### 3.1. Toll-Like Receptors (TLRs)

TLRs are the key components of innate immunity that are evolutionary conserved [52]; they are involved in early interactions with invading microorganisms by detecting microbial pathogen-associated molecular patterns [53]. TLRs are type I transmembrane proteins, and they have a conserved structure with an N-terminal ectodomain containing leucine-rich repeats, a single transmembrane domain, and a cytosolic TIR domain [54]. TLRs play an important role in early host defense by initiating the innate immune response and shaping the adaptive immune response that limits the spreading infection [55,56]. TLRs from each family exert similar functions across species [57,58]. TLRs are encoded by a large gene family, and the TLR family comprises 10 members (TLR1–TLR10) in humans and 12 (TLR1–TLR9, TLR11–TLR13) in mice [59]. So far, 10 TLRs (TLR2–10 and TLR13) have been reported in koalas [60]. TLRs can be classified into cell-surface TLRs and intracellular TLRs based on their localization. TLR1, TLR2, TLR4, TLR5, TLR6, and TLR10 are cell-surface TLRs, whereas TLR3, TLR7, TLR8, TLR9, TLR11, TLR12, and TLR13 are intracellular TLRs that are localized in the endosome [61,62]. However, on the basis of functional roles, TLRs can be sub-divided into viral and non-viral TLRs. Viral TLRs include TLR3, TLR7, TLR8 and TLR9; TLR3 is known to recognize dsRNA and DNA viruses [63], TLR7 and TLR8 recognize ssRNA [64], and TLR9 recognizes unmethylated CpG-containing DNA that is usually present in the genomes of DNA viruses [65]. Non-viral TLRs such as TLR1, TLR2, TLR6 and TLR10 can respond to lipopeptide from bacteria and parasites [66,67], TLR4 recognizes lipopolysaccharides (LPS) from Gram-negative bacteria [68], TLR5 recognizes flagellins [67], and TLR13 recognizes bacterial 23S ribosomal RNA [69].

Although TLRs are the most widely characterized PRRs, our understanding of the role of TLRs in koala immunity is limited. Cui et al. characterized genetic polymorphisms in 20 wild koalas, and found genetic polymorphisms in all koala TLR genes, except TLR10, and a total of 40 single nucleotide polymorphisms (SNPs) were also identified across all loci [60]. It has been reported that genetic variations, including SNPs, may greatly influence innate immune responses against invading pathogens, and also disease outcomes [70,71]. Further study to detect the role of the identified SNPs in different KoRV subtypes is warranted. Several retroviruses have been identified in many vertebrates [72], and retroviruses can be detected by several TLRs, which should contribute to the anti-retroviral response [73]. However, TLR response is critical, which may act as a double-edged sword in protecting against pathogens or the induction of immune-mediated pathological consequences in the host [74,75,76]. Although it is believed that TLR response is crucial for the anti-retroviral immune response, much remains to be known about the mechanisms of TLR interactions by which TLR stimulation can inhibit or enhance retroviral infections.

**Figure 1:**
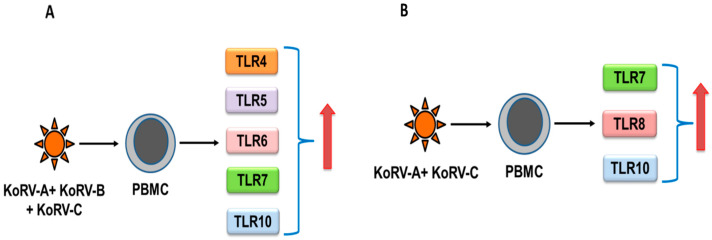
TLR response to infection with multiple KoRV subtypes in koalas (summarized from [77]). When compared with KoRV-A-infected koalas, upregulation was observed in TLR4-TLR7, and TLR10 mRNA in KoRV-A, KoRV-B, and KoRV-C-infected koala peripheral blood mononuclear cells (PBMCs) (**A**), and TLR7, TLR8, and TLR10 mRNA in KoRV-A and KoRV-C–infected koala PBMCs (**B**).

Recently, we characterized TLR expression patterns in koala PBMCs, and observed that all of the reported TLRs (TLR2–10 and TLR13 mRNA) were expressed in PBMCs [77]. The expression of TLR4–7 and TLR10 were upregulated (Figure 1A) in koalas infected with endogenous KoRV-A and exogenous KoRV-B and KoRV-C, compared to KoRV-A–only infected koalas, and TLR7, TLR8, and TLR10 expressions were also upregulated (Figure 1B) in koalas infected with endogenous KoRV-A and exogenous KoRV-C, compared to KoRV-A–only infected koalas, which may imply the role of TLRs in KoRV infection [77]. However, this study was limited by sample size, where ten koalas were used in the absence of any KoRV-negative koalas. Moreover, whether these have significant downstream effects on signaling pathways in koalas is yet to be characterized. Differential expression of TLRs were also observed in tissues obtained from two koalas [77]. However, the phenotype of TLR expression also needs to be investigated in KoRV-negative and KoRV-positive koalas with multiple KoRV subtypes from a range of habitats and disease states in order to gain further insights into the future exploitation of TLR modulators [78].

### 3.2. Cytokines

**Figure 2:**
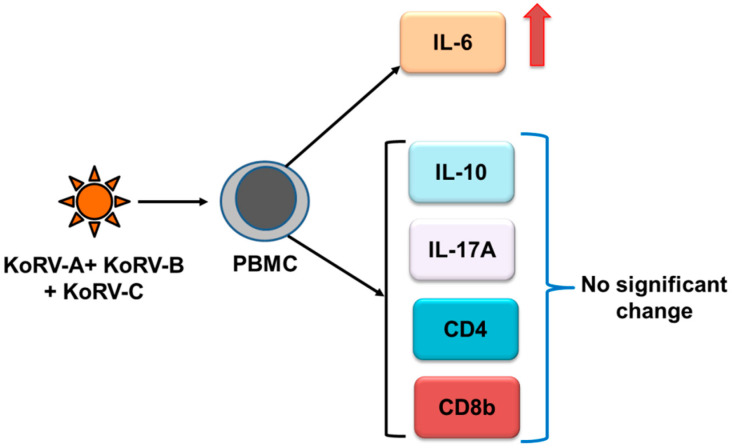
Cytokine, CD4, and CD8b responses to multiple KoRV subtype infections in koala PBMCs (summarized from [84]), which were compared to KoRV-A–only infected koalas.

Cytokines are critical mediators that are required to establish communication in the host immune system and play a crucial role in host defense against pathogens [79]. Cytokine production may vary depending on the virus and cell type [80,81]. Immunological studies are essential for the proper understanding of the host immune response to a particular pathogen in order to devise prophylactic and therapeutic interventions against disease [82,83]. Although much remains to be known about the immunomodulatory effects of KoRV on koalas, the recent advancement of koala genetic resources should greatly facilitate immune response studies in this marsupial species. The first evidence for the immunomodulatory effects of KoRV was obtained by the increased expression of interleukin-6 (IL-6), IL-10, growth-related oncogene and monocyte chemotactic protein-1 upon the exposure of KoRV to human PBMCs [40]. However, the advances of CD4, CD8, and cytokine response in KoRV infection have been described in recently published reviews [2,22]. Here, we will discuss the cytokine response to multiple KoRV subtype infections, including endogenous and exogenous subtypes. In our recent study, we found a significantly upregulated expression of IL-6 in koala PBMCs infected with two exogenous KoRV subtypes (KoRV-B and KoRV-C) in addition to the endogenous KoRV subtype (KoRV-A) compared to koalas with only the endogenous (KoRV-A) infection; however, no significant change in expression was observed for CD4, CD8b, IL10, and IL-17A (Figure 2) [84]. However, this study was limited by sample size, where nine koalas were used, and no KoRV-negative koala could be included in that study. Moreover, the phenotype of cytokine expression needs to be investigated in a large number of koala populations, where KoRV-negative and KoRV-positive koalas with multiple KoRV subtypes from a range of habitats and disease states will be included for a clear picture of cytokine interactions in KoRV infection in koalas.

## 4. Maintenance of Koala Health and Conservation by Vaccine Development and the Investigation of Alternative Strategies

The majority of Australia’s koalas are infected with KoRV [2], which has been linked to several life-threatening diseases, including lymphoma and leukemia, and also chlamydial infections. Therefore, koala health and conservation are under a big challenge, and koalas have been listed as vulnerable on the International Union for Conservation of Nature (IUCN) ‘red list’ of threatened species [85]. Until now, vaccines have been one of most successful medical interventions for protection against infectious and viral diseases [86,87]. While different measures such as quarantine and the use of antiretroviral drugs are possible for controlling KoRV, they are impractical, and vaccination is the realistic option [88]. Moreover, currently, there are no treatments available for KoRV in koalas. Also, until now, no licensed vaccines have been commercially available for KoRV. However, vaccine development remains the best choice for a KoRV management strategy, and scientists’ efforts are ongoing toward the development of KoRV vaccines to successfully intervene in KoRV infection in koalas. However, little has been discovered about the dynamics of the innate immune response in the context of various KoRV subtype infections. Furthermore, the potential role of the innate immune response in vaccine efficacy remains to be investigated. Below, we discuss the recent advances of KoRV vaccine development and their response in koalas.

### 4.1. Vaccine Response in Koalas against KoRV

**Figure 3:**
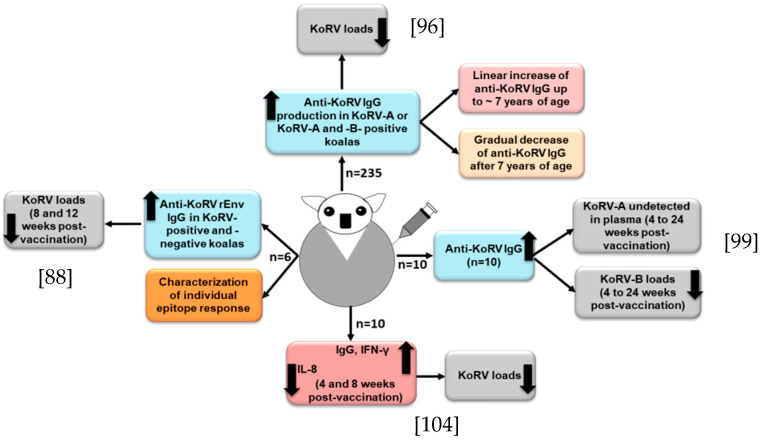
Cartoon shows KoRV vaccine responses in KoRV-infected koalas. In each case [88,96,99,104], anti-KoRV IgGs were developed and viral loads were reduced.

For the maintenance of koala health and conservation, effective prophylactic vaccines against the exogenous KoRV subtypes are in urgent need of development. Although it is challenging to develop a successful vaccine against a retrovirus [89], an effective vaccine against FeLV (a gammaretrovirus) infection has been developed [90], which may suggest that vaccine development for KoRV might be feasible. However, during the exploration of the antibody response, Fiebig et al. found a lack of antibody response in 16 northern koalas, which might suggest a state of tolerance in those koalas [91]. Towards a continued effort of developing a prophylactic KoRV vaccine, Fiebig et al. observed that a recombinant KoRV (rKoRV) Env protein could induce neutralizing antibodies in rats and goats [92]. In a subsequent study with three northern koalas, a recombinant KoRV-A Env vaccine induced Env antibodies, and the vaccinated koalas did not show any sign of adverse reactions [93]. In another study, Olagoke et al. selected recombinant KoRV envelope protein (rEnv) as the vaccine antigen by epitope mapping analysis, which consisted of a segment of KoRV env protein and a synthetic membrane proximal external region (MPER) peptide [88]. MPER is considered to be an attractive vaccine target due to its conserved nature and induction of broadly neutralizing antibodies (bNAbs) [94,95]. The rEnv -based anti-KoRV vaccine induced a significant humoral immune response and neutralizing antibodies in both KoRV-positive (KoRV-A positive, but KoRV-B negative) and KoRV-negative koalas [88]. In order to further investigate, Olagoke et al. performed a large-scale study with 235 wild koalas harboring KoRV-A with or without KoRV-B, and found the production of anti-KoRV antibodies in about 95% of the vaccinated koalas [96] (Figure 3); antibody production against endogenous KoRV could partly explained by the role of Piwi-interacting RNAs (piRNAs) in koalas [17]. There was an association between koala age and the production of anti-KoRV IgG levels upon vaccination, where a linear increase of anti-KoRV IgG level was observed up to 7 years of age, and there was a gradual decrease of anti-KoRV IgG level after 7 years of age (Figure 3) [96], which could be partly explained by the reduction of the functional innate immune cell numbers that are related to aging [97].

It is notable to mention that, in our recent study, we observed a high KoRV RNA load in the plasma of one koala, and when it was tested again after several months, that koala became negative for KoRV [86], which might be due to the induction of the immune response that finally cleared the KoRV from the plasma [98]. Olagoke et al. further reported that therapeutic vaccination with a recombinant KoRV Env protein combined with a Tri-adjuvant in koalas infected with endogenous retrovirus induced anti-KoRV IgG and neutralizing antibodies and resulted in a complete clearance of KoRV-A from the plasma. Moreover, there was a decreased KoRV-B viral RNA level in plasma, which is suggestive of the induction of cross-reactive antibodies [99] (Figure 3). Its efficacy against other exogenous KoRV subtypes should also be investigated.

A proper understanding of koala’s immune response against KoRV is a key step in therapeutic and prophylactic interventions for better conservation and management strategies to protect koalas. Moreover, deciphering the role of TLRs in the adaptive immune response could be an important area of future study [100]. A balanced cellular and humoral immune response is essential for preventing retrovirus infection [89]. It has been reported that both the innate and adaptive immune response play important roles in the control of ERVs in mice [101,102]. The innate immune response induces a rapid inflammatory cytokine burst and activates antigen-presenting cells, which play an important role in the conditioning of the immune system for the further development of a specific adaptive immune response [103]. A recent study investigated the expression profiles of key immune genes upon vaccination, and observed a significant upregulation of interferon-γ (IFN- γ) and decreased IL-8 expression (Figure 3); however, no significant changes in the mRNA expression of CCL4L, IL-1β, IL-10, IL-18, IL-4, and IL-6 were observed at either 4- or 8-weeks post-vaccination [104]. Both the cell-mediated and humoral immune response were induced, and viral loads were decreased [104]. However, the koalas used in that study had very low CD4 levels compared to their CD8β levels, which might be linked to KoRV infection; however, the vaccination did not improve the condition, which is important to investigate in a future study. Olagoke et al. also observed an expression of four host restriction factors, such as BST2, ISG15, RSAD2 and TRIM1, in endogenous KoRV harboring koalas, with a relatively higher expression of BST2, ISG15 and RSAD2, which is suggestive of the biological importance of these restriction factors in koalas [104]. Different restriction factors have been identified in humans and animals that are known to inhibit virus replication [105]. However, the roles of these restriction factors in koalas remain to be investigated.

Although there have been many advances towards vaccine development, the efficacy of the KoRV vaccines against multiple KoRV subtype infections, including KoRV C to J, remains to be investigated. Moreover, it has been reported that purified TLR agonists could be used as adjuvants in vaccination to enhance the adaptive response [106], which could be investigated in koalas, particularly where the antibody response is poor or lacking [91]. However, the development of an effective pan-subtypic KoRV vaccine is a major area of interest for protecting koala health. Moreover, the advances of KoRV vaccine development would benefit the field of other retrovirus vaccine development.

### 4.2. Alternative Strategies to Control KoRV Infection

It is of great importance to investigate for alternative strategies to avoid KoRV threats to koala health and conservation. piRNAs are a distinct class of small non-coding RNAs, which are 24–31 nucleotides in length and can silence transposable elements, regulate gene expression, and fight viral infection [107,108]. In order to prevent deleterious germline mutations, it is important to suppress the active replication of ERVs and virus-like elements in the germ line. However, ERV and retrotransposon activities are not limited to the germline only; they also occur in somatic tissues and can induce disease [109,110,111]. PiRNAs play an important role in the maintenance of genomic stability of many animal species. piRNAs form the piRNA-induced silencing complex (piRISC) in the germ line, and they protect the integrity of the genome from invasion of transposable elements (TEs) by silencing them [112,113].

piRNA-mediated silencing has been shown to be important for the repression of TEs activity in mouse germ cells [112]. Therefore, it is of great interest to investigate the evolutionary control of KoRV infection in koalas by characterizing piRNAs. The piRNA pathway is a conserved defense mechanism that functions as a safeguard of genome integrity and the fertility of animal germ cells [114]. In a recent study, Yu et al. characterized koala piRNAs, and transposon-mapping revealed that piRNAs in koala are roughly equally sense- and antisense-oriented, with a typical 1U and ping-pong signatures [17]. They found that KoRV-A and three endogenous retrotransposons are active in the koala germline and soma; however, their activities were lower in the testis compared to somatic tissues, where the piRNA pathway is active [17]. They also observed that unspliced KoRV-A proviral transcripts are preferentially processed into piRNAs, and the piRNA emerging from unspliced transposon transcripts is deeply conserved [17]. More importantly, in that study, they indicated the role of piRNAs in responding against KoRV germline invasion, where both the innate and adaptive phases of piRNA response were generated [17]. However, in order to gain further insights into KoRV-A association in endogenous transposon mobilization, KoRV-free animals could be investigated, which are present in the southern end of the koala habitat [22].

Recently, Tarlinton et al. reported that koalas may maintain defective KoRV as protection from the infectious form of KoRV, and those koalas could be selectively used for breeding in order to avoid the threat of infectious KoRV [115]. CRISPR/Cas9 appears to be a revolutionary genome editing tool which could be used in the control of viral infections [116]. Recently, Yang et al. succeeded in inactivating PERV in a porcine kidney epithelial cell line (PK15) using CRISPR-Cas9 by targeting the PERV pol gene [117]. In a subsequent study, Niu et al. became able to inactivate all of the PERVs in a porcine primary cell line using CRISPR-Cas9 technology, and developed PERV-inactivated pigs via somatic cell nuclear transfer [118]. Using a combination of CRISPR-Cas9 and transposon technologies, Yue et al. performed an extensive germline genome engineering in pigs, and showed the inactivation of all the PERV in pigs [119]. These findings also show the suitability of utilizing CRISPR/Cas9 technology to control KoRV infection in koalas, and future study in this regard is warranted.

## 5. Conclusions

The understanding about the interactions of KoRV and its host is advancing. However, a thorough study of the host immune response against multiple KoRV subtypes could aid further insights into the understanding of the potential of KoRV in the host’s immunopathogenesis, as well as prophylactic and therapeutic KoRV vaccine development, which will be a big step towards koala health and conservation strategies. Moreover, the deep transcriptome analysis of koala populations from different geographic locations is of great importance to further deciphering any underlying genetic components’ effects on koala health and conservation [120].
